# *Expect With Me*: development and evaluation design for an innovative model of group prenatal care to improve perinatal outcomes

**DOI:** 10.1186/s12884-017-1327-3

**Published:** 2017-05-18

**Authors:** Shayna D. Cunningham, Jessica B. Lewis, Jordan L. Thomas, Stephanie A. Grilo, Jeannette R. Ickovics

**Affiliations:** Yale School of Public Health, 135 College Street, Room 226, New Haven, CT 06510 USA

**Keywords:** Implementation study, Pregnancy, Group prenatal care, Preterm birth, Innovation

## Abstract

**Background:**

Despite biomedical advances and intervention efforts, rates of preterm birth and other adverse outcomes in the United States have remained relatively intransigent. Evidence suggests that group prenatal care can reduce these risks, with implications for maternal and child health as well as substantial cost savings. However, widespread dissemination presents challenges, in part because training and health systems have not been designed to deliver care in a group setting. This manuscript describes the design and evaluation of *Expect With Me*, an innovative model of group prenatal care with a strong integrated information technology (IT) platform designed to be scalable nationally.

**Methods/Design:**

*Expect With Me* follows clinical guidelines from the American Congress of Obstetricians and Gynecologists. *Expect With Me* incorporates the best evidence-based features of existing models of group care with a novel integrated IT platform designed to improve patient engagement and support, enhance health behaviors and decision making, connect providers and patients, and improve health service delivery. A multisite prospective longitudinal cohort study is being conducted to examine the impact of *Expect With Me* on perinatal and postpartum outcomes, and to identify and address barriers to national scalability. Process and outcome evaluation will include quantitative and qualitative data collection at patient, provider, and organizational levels. Mixed-method data collection includes patient surveys, medical record reviews, patient focus groups; provider surveys, session evaluations, provider focus groups and in-depth interviews; an online tracking system; and clinical site visits. A two-to-one matched cohort of women receiving individual care from each site will provide a comparison group (*n* = 1,000 *Expect With Me* patients; *n* = 2,000 individual care patients) for outcome and cost analyses.

**Discussion:**

By bundling prevention and care services into a *high-touch, high-tech* group prenatal care model, *Expect With Me* has the potential to result in fundamental changes to the health care system to meet the “triple aim:” better healthcare quality, improved outcomes, and lower costs. Findings from this study will be used to optimize the dissemination and effectiveness of this model.

**Trial registration:**

ClinicalTrials.gov, NCT02169024. Retrospectively registered on June 18, 2014.

## Background

Birth outcomes are a nationwide health improvement priority [1]. Nearly four million women in the United States give birth each year, and 84% will give birth in their lifetimes [2, 3]. Despite substantial biomedical advances and intervention efforts, rates of preterm birth (9.6%) and low birthweight (8.1%) in the United States have remained relatively intransigent over the past three decades [4] and are considerably higher than rates in all other developed nations [5]. Extreme racial and ethnic disparities persist in the prevalence of preterm birth and low birthweight as well as consequent infant mortality. Compared to non‐Hispanic White women, Black women are about 50% more likely to deliver preterm and 90% more likely to deliver a low birthweight infant [4]. Infant mortality due to preterm or low birthweight is 3.5 times higher for Black women and nearly two times higher for Puerto Rican women, compared to non-Hispanic White women [6].

The financial and human costs associated with preterm and low birthweight babies are profound. These adverse birth outcomes account for 36% of all US infant deaths [7], and are associated with greater infant and childhood morbidity as well as increased health care costs. Complications of preterm birth include neurodevelopmental disabilities, school and behavioral problems, visual and hearing impairment, cardiovascular and metabolic disorders, and higher risk of preterm in the next generation [8, 9]. Low birthweight is associated with subsequent risk of coronary heart disease, hypertension, and diabetes [10], and has been linked to all-cause mortality among women, and premature death among men [11]. In 2005, the annual economic burden associated with preterm birth in the US exceeded $26 billion [12]. Based on health care inflation from 2005 to 2015 [13], we estimate that these costs now would exceed $38 billion. Employers pay an average of twelve times more for newborn medical care for a preterm birth than for a healthy, full-term baby in the first year of life [14]. Families incur direct medical and non-medical (e.g., special education) costs, as well as indirect costs (e.g., lost productivity). Moreover, preterm birth and low birthweight result in substantial emotional distress for parents who must advocate and make healthcare decisions for fragile newborns [15].

Preventive interventions to address preterm and low birthweight include quality improvement efforts to eliminate early elective deliveries, smoking cessation, limiting multiple embryo transfer for in-vitro fertilizations, progesterone therapy to help sustain pregnancies among women with prior spontaneous preterm birth, and cervical cerclage for women with short cervical lengths [16]. However, the causes of preterm birth are not well understood, and as many as two-thirds of preterm births have unknown etiologies [17]. Group prenatal care, involving shared medical visits among pregnant women, has been suggested as one way through which substantial improvements in birth outcomes may be achieved [18–21].

In group prenatal care, a credentialed prenatal provider (e.g., obstetrician, midwife) provides pregnancy care to eight to twelve women simultaneously during up to ten 90–120 min group visits, and follows American Congress of Obstetrics and Gynecology (ACOG) guidelines. These group sessions integrate pregnancy health assessments with additional education, skills building and peer support.

A recent meta-analysis concluded that group prenatal care was associated with a decreased rate of low birth weight overall and a reduction in risk for preterm birth for African American women, compared to individual care [22]. Significant clinical, methodological, and statistical heterogeneity between available studies limited the authors’ ability to assess other outcomes. However, the studies Carter and colleagues deemed to be the most rigorous (i.e., two NIH-funded randomized controlled trials from our research group) documented that, compared to standard individual care, group prenatal care results in lower rates of preterm and small for gestational age babies, less incident sexually transmitted infections, healthier maternal weight trajectories, and fewer depressive symptoms as well as increased patient satisfaction with care [18, 21, 23–25]. These improved outcomes may be due to reduced stress and increased knowledge, motivation, and health care engagement, resulting from the additional education and support that group prenatal care patients received [26]. More research is needed to replicate the effects of group prenatal care on perinatal and postpartum outcomes, and to identify potential mechanisms through which group prenatal care impacts health.

Estimated cost savings associated with group prenatal care (due to improved outcomes) varies by geography, ranging from $750 to $890 per birth [27]. Currently, such savings are realized by payors, such as Medicaid and private health insurers—not clinical health systems providing care. According to analyses conducted by Optum (UnitedHealth Group), if one-half of pregnant women enrolled in Medicaid received group prenatal care, net savings to Medicaid would be approximately $12 billion over the next decade [28]. A recent study aimed at determining Medicaid costs savings associated with a group prenatal care program in South Carolina documented a $2.3 million return on investment, with an average savings of $22,667 in health care expenditures for every preterm birth prevented [29].

Despite the potential for improved outcomes, group prenatal care currently is available to an estimated 3% of pregnant women in the United States [30]. Although evidence suggests that, if given a choice, approximately 50% of women would choose to participate in group care [31], widespread dissemination of group prenatal care presents challenges. First, any disruptive innovation is likely to face challenges and resistance from complex systems, such as the healthcare system, which seek homeostasis [32]. Second, the healthcare system was not designed to provide patient care in groups (i.e., infrastructure: group space, information technology systems for patient scheduling, provider scheduling, charting). Providers have not been trained to deliver care in groups (e.g., group facilitation skills, providing 20 h of patient education per pregnancy). Providers are conditioned to keep new patients for continuity of care, not refer them to the provider starting the next new prenatal group, where they will receive the remainder of care (e.g., loss of billable hours, turf issues).

Transitioning a health system from individual to group prenatal care is a challenging task that requires an organizational culture that supports innovation, one or more champions who will lead change efforts, and buy-in from administrators, clinicians, and staff [33, 34]. Moreover, a financial paradox exists whereby the healthcare delivery system bears the burden of transformation to provide group care, yet it is often not the financial beneficiary of outcome improvements. Often, prenatal clinics pay start-up (e.g., training) and ongoing (e.g., materials, accreditation) costs to deliver group prenatal care. However, much of the cost savings come from averted (or shorter) neonatal intensive care unit stays or reduced emergency department visits; those savings are not channeled back to prenatal clinics, but rather are realized by other departments or payors.

To promote more widespread adoption and sustainability of group prenatal care, we developed an innovative model of group prenatal care with a novel information technology (IT) platform, called *Expect With Me. Expect With Me* group prenatal care was designed to be scalable nationally at lower cost to clinical practices and healthcare systems through the use of technology and the engagement of payors. Since February 2014, *Expect With Me* has been implemented with more than 1,000 women in five sites in Nashville TN, Detroit MI, and McAllen TX. This paper describes the design of the intervention and evaluation plan to assess its implementation and impact on health care quality, outcomes, and costs.

## *Expect With Me* group prenatal care

*Expect With Me* group prenatal care was designed based on: (1) principles of group care [35]; (2) evidence from randomized controlled trials demonstrating improved birth outcomes for women receiving prenatal care in a group format [18, 21]; (3) clinical guidelines for prenatal care delivery [36]; and (4) research on patient and provider engagement through social media and technology [37–39]. Designed with national dissemination as a primary consideration, *Expect With Me* incorporates the best evidence-based aspects of existing models of group care [22, 40–42] with a novel IT platform to improve patient engagement and support, enhance health behaviors and decision making, connect providers and patients, and improve health service delivery.

*Expect With Me* provides care to groups of 8–12 women of the same gestational age. Women receiving *Expect With Me* begin prenatal care in the traditional manner. Formal intake (history, exam) is performed at an initial visit prior to group assignment. All prenatal care thereafter occurs within a group setting, except for health issues that require privacy and cervical assessments in late pregnancy. Ultrasounds and laboratory screenings occur per ACOG clinical guidelines [36]. *Expect With Me* is implemented from week 14 of pregnancy (after initial individual assessment) through delivery, following the same schedule as individual care. However, group visits are 90–120 min each, and follow a unique structured curriculum that incorporates the standard content of prenatal care, and emphasizes critical contemporary health issues relevant to pregnancy, such as nutrition, physical activity, stress/mental health, and sexual health. Table 1 summarizes the timing and recommended topics to be covered during each session. Participants may bring their partner, family member, or other support person to group sessions.

**Table 1:** *Expect With Me* group prenatal care session timing and topics

Session	Themes	Topics
1 (13–17 weeks)	You’re a healthy mom	• Eat and live healthy for you and your baby • Stay active while you’re expecting • Maintain healthy weight during pregnancy • Understand routine prenatal testing and emergencies • Know what blood pressure and weight numbers are healthy for you
2 (17–21 weeks)	Staying healthy and strong through change	• How babies grow and develop • Mom’s clean teeth = healthier mother and baby • Learn why you’re feeling the way you do • Move safely and comfortably while pregnant • Get a good night’s sleep • Keep calm and stress-free while expecting • Stay safe at home, work and play
3 (21–24 weeks)	Breastfeeding = Healthy Babies and Healthy Moms	• Benefits of breastfeeding • Barriers to breastfeeding • Basics of breastfeeding • Choose a pediatric provider (Part 1) • Your support systems (Part 1)
4 (25–29 weeks)	Healthy moms building healthy relationships	• Understand Gestational Diabetes Testing • Build healthy relationships • Prevent STDs including HIV (Part 1) • Choose when to get pregnant (Part 1)
5 (27–31 weeks)	Healthy moms and healthy labor	• Signs of labor • Stages of labor (Part 1) • Fetal heart rate monitoring • Stay comfortable during labor • Understand Cesarean birth
6 (29–33 weeks)	Healthy labor	• Stages of Labor (Part 2) • What happens immediately after delivery • Labor and delivery decisions • Provider policies and options for labor and delivery • Prevent STDs including HIV (Part 2)
7 (31–35 weeks)	Healthy labor and healthy relationships	• Prepare for hospital stay and return home • Negotiate to build healthy relationships • Understand Group B Strep testing and prevention
8 (33–37 weeks)	Taking care of mom and baby	• Caring for your baby • Choose a pediatric provider (Part 2) • Care for your postpartum body • Set goals to build healthy relationships (Part 1)
9 (35–39 weeks)	Preparing for a Healthy Future	• How to breastfeed • Staying healthy and strong after pregnancy • Signs of postpartum depression • Make sure your home is safe you and your baby
10 (37+ weeks)	Build a healthy future	• Choosing a daycare provider • Going back to work • Your support systems (Part 2) • Choose when to get pregnant again (Part 2) • Set goals for a healthy relationship (Part 2)

In a group setting, credentialed prenatal providers (e.g., obstetrician, midwife) conduct one-on-one assessments with each patient (30 min) and then facilitate group discussions on the topics of pregnancy, using adult learning principles (60–90 min). Facilitated discussions allow patients to provide and receive peer support while gaining knowledge and skills related to explicit learning objectives on pregnancy, childbirth, and parenting [43]. *Expect With Me* meets a broader set of needs for pregnant women (e.g., medical, social, educational) than traditional care; yet, it is fully reimbursable by health insurance programs as prenatal care. Further, women access the IT platform during their prenatal visit to track their own health metrics (e.g., weight, blood pressure, visit attendance). This encourages patient engagement in self-care and introduces them to the online experience of care that will continue throughout their pregnancy and postpartum.

**Fig. 1:**
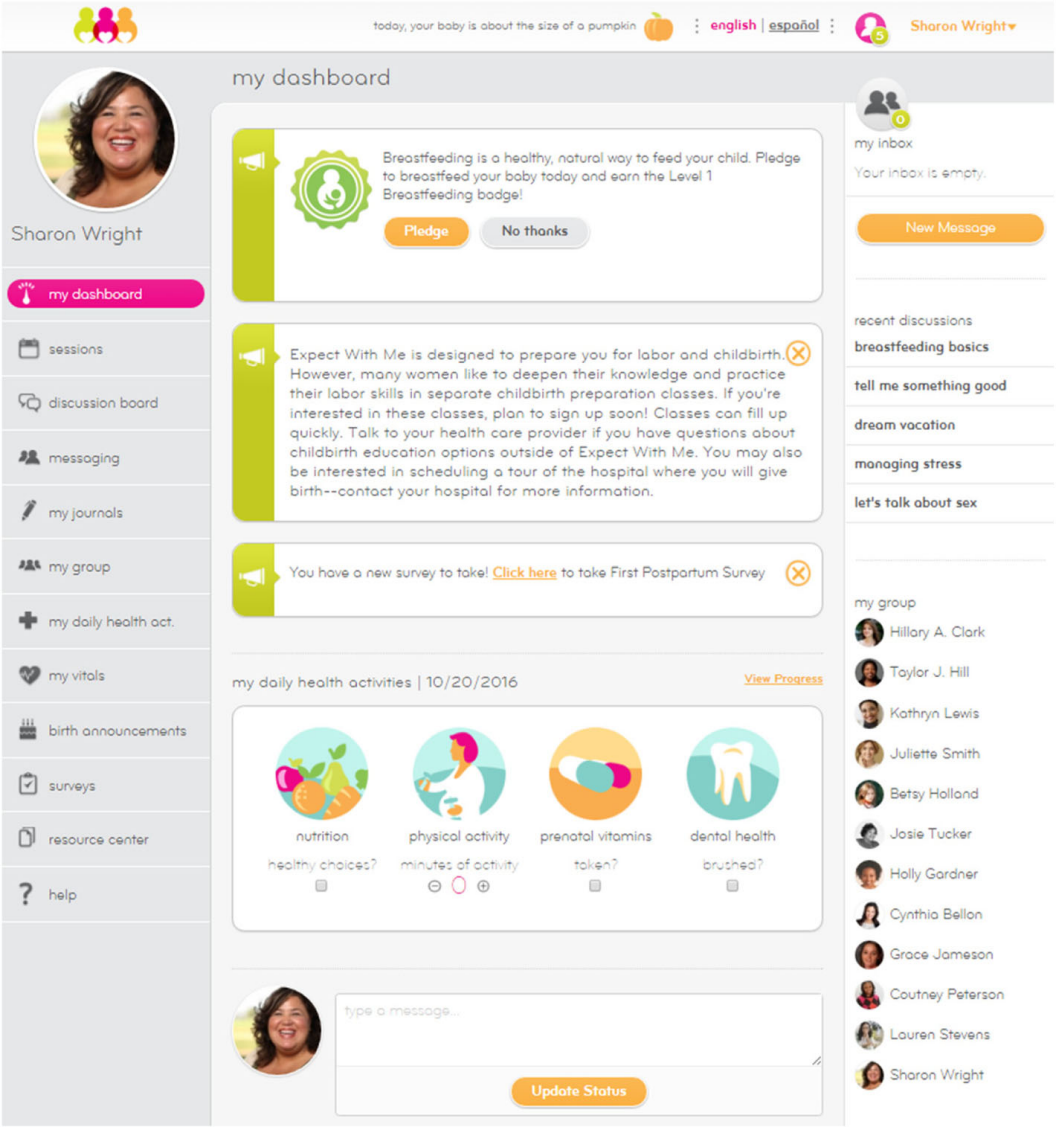
*Expect With Me* IT platform: Select patient views (all patient names and photographs are fictional)

*Expect With Me* has a novel, HIPPA-secure, integrated IT platform that enables patients to track their own health metrics, communicate with health providers and fellow group patients, access healthcare resources and educational materials, provide and gain support (Fig. 1). It is optimized for use on smartphones and computers, with all content available in English and Spanish via a single toggle. Personalized profiles allow patients to log vital signs (i.e., blood pressure, weight) during prenatal visits and to view their own weight trajectory across pregnancy. It also prompts them to track health behaviors (e.g., taking prenatal vitamin, drinking water, exercising) when they log in. Patients can access educational materials, including videos, tip sheets, audio files with relaxation/mindful meditation exercises, and links to online resources. Women can journal about their pregnancy experience, send messages to other women in their group, participate in discussion board conversations, and send out birth announcements.

**Fig. 2:**
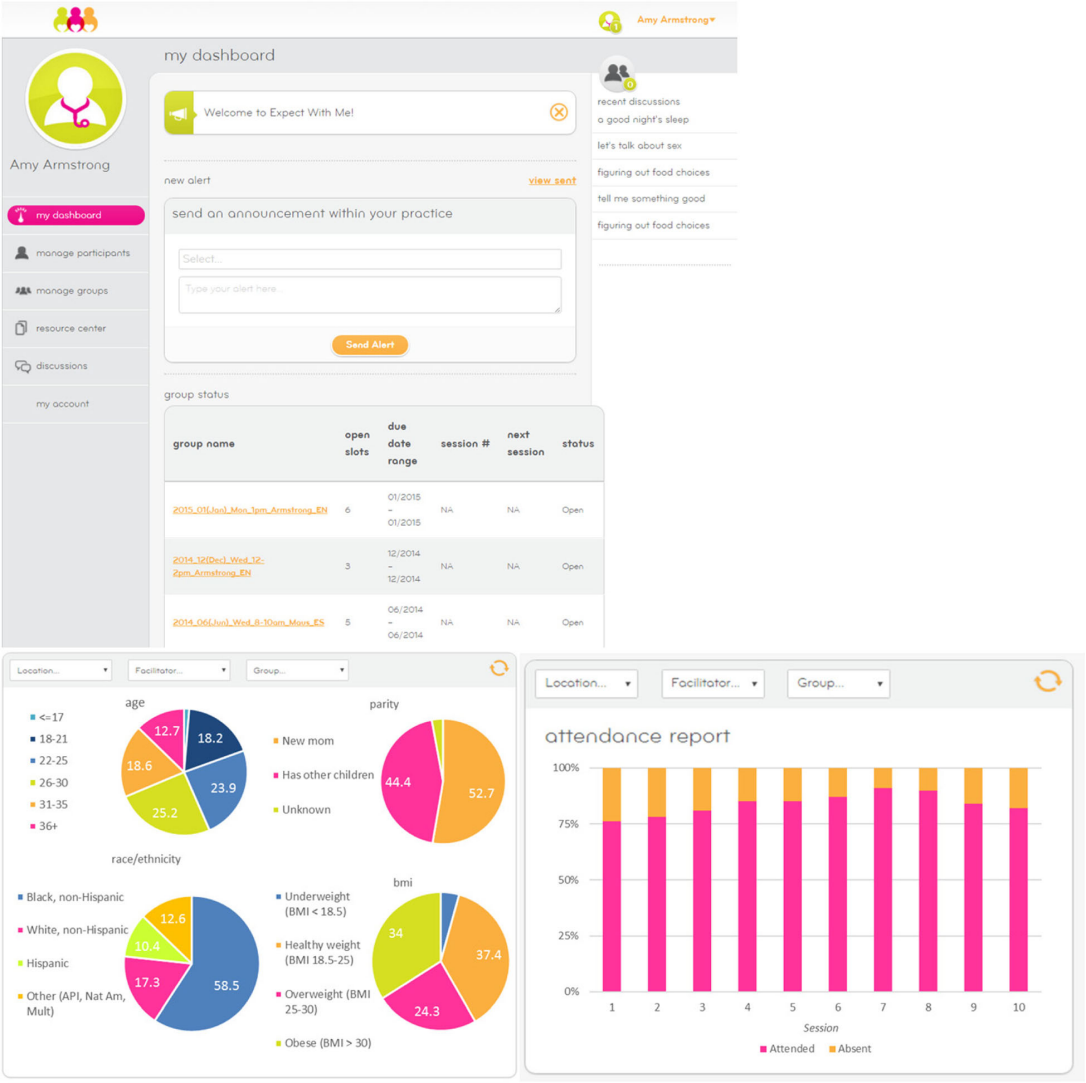
*Expect With Me* IT platform: Select clinician views

*Expect With Me’s* integrated information technology platform has numerous features to ease clinical implementation and aid practice management. All facilitator and patient handbooks are available electronically in both English and Spanish, eliminating the need for costly printing of materials. Prenatal care providers can use the IT platform to monitor attendance, upload and distribute educational materials to patients between visits, document care/content delivered, identify patient needs, and plan targeted care, such as inclusion of an HIV counselor or nutritionist in the next session. A scheduling tool is available to aid the establishment group care schedules that account for provider time, group space, clinic schedules, holidays and more. Health systems have access to real-time data on patient demographics and adherence, to monitor and evaluate implementation success (Fig. 2).

## Development of *Expect With Me*: theoretical basis and formative research

*Expect With Me* was developed by a transdisciplinary team of researchers at Yale University, representatives from UnitedHealth Group, and health care providers at Vanderbilt University Medical Center. It is based on principles of group care, which assert that care is most effectively and efficiently provided in groups, that learning and support are enhanced, and that this high quality of care is difficult to achieve within the traditional structure of individual examination room visits [35, 44, 45]. Group prenatal care provides substantially more contact with providers (from two hours across pregnancy in individual care to 20 h in group), provides support services, and is integrated to respond to complex needs of pregnant women. Advantages of group interventions include, but are not limited to: improved learning and skills development, attitude change and motivation, enhanced insight through sharing of common experiences, and social support [43, 46]. In turn, groups facilitate development of new community norms for health-enhancing behaviors.

*Expect With Me* was designed using a human-centered design approach. Human-centered design is an iterative and participatory process to help ensure innovations are acceptable, usable, and meet the needs of users [47]. This approach has been used to shape healthcare to meet the multiple levels of need of clinicians and patient populations [48–51]. A series of key informant interviews with twenty prenatal care stakeholders and observational data collection during prenatal care visits were conducted, from which two important findings emerged. First, the healthcare industry historically has defined prenatal care in medical terms, and an important value proposition of group prenatal care is that it provides a context in which other basic, safety, and social needs can be addressed. Second, pregnancy provides an opportunity to re-evaluate how those needs are being met, and prenatal care messaging would benefit by moving from a frame of fear and avoidance (e.g., what not to eat, drink, and do) to embracing good health habits for women and their families. An independent team of four curriculum development and writing professionals were commissioned and—following clinical guidelines from the American Congress of Obstetricians and Gynecologists and best practices for group facilitation—developed a ten-session structured curriculum and supplemental materials for the IT platform, grounded in the lessons learned during this process. To ensure accuracy, relevance, and feasibility, all content associated with *Expect With Me* (e.g., facilitator and patient handbooks, tip sheets and other resources) was reviewed by a medical team, including obstetricians/maternal-fetal medicine specialists, midwives, and pediatricians). A pilot study of *Expect With Me* was conducted with 243 pregnant women in Nashville TN; based on this pilot, revisions were made to enhance structure and content of the curriculum. The result of this extensive formative research is a supportive, comprehensive model of group prenatal care that aims to identify and address the full spectrum of pregnant women’s needs throughout pregnancy and beyond, fostering long-term health for women and their families.

## Methods/Design

*Expect With Me* is being evaluated via a multisite, longitudinal matched-cohort study, in which data are collected in a real-world settings at the patient and organizational levels. For national scale-up and sustainability, it is essential that the program not only be effective for patients but that the implementation strategy fits with the context of health clinics providing prenatal care [52]. A rigorous multi-method evaluation is being conducted to examine the impact of *Expect With Me* on maternal health and birth outcomes and identify and address barriers to national scalability. Cost analyses are also planned.

The study includes a rigorous process and outcome evaluation. Birth outcomes are the primary study outcomes (e.g., preterm birth, birthweight, neonatal intensive care unit admission/duration). Maternal psychosocial, health behaviors, and health outcomes (e.g., depression, breastfeeding, postpartum weight loss) are secondary outcomes.

The process evaluation will identify factors that influence uptake, fidelity, and sustainability of *Expect With Me* to inform scalability. We will employ quantitative approaches, such as online surveys and health record reviews as well as systematic qualitative approaches, such as in-depth interviews and focus groups. Key components of comprehensive process evaluations specified by Steckler and Linnan [53] will be adapted to meet the specific needs of this study. Uptake will include reach (number of groups offered); dose delivered (extent to which providers enroll patients and facilitate *Expect With Me* sessions); and dose received (willingness of eligible patients to participate; attendance in group sessions). Fidelity refers to the quality and integrity of the intervention as conceived, and is a function of implementation by clinical sites. We will examine how groups were planned and populated, the content and process of each session, and the perceptions of site staff and patients about *Expect With Me*. We will document whether *Expect With Me* patients received the intended exposure to the intervention, including attendance at each session; proportion of prenatal visits delivered in group; and patient use of IT platform. Sustainability will be driven, in part, by the impact of *Expect With Me* on healthcare costs. We will analyze the impact of birth outcomes on healthcare costs. We will document differences in utilization of care (e.g., emergency department, neonatal intensive care unit) between *Expect With Me* and individual care. Sustainability is impacted by depth of training and satisfaction with *Expect With Me* by providers and staff. We will document penetration of staff trainings and collect feedback from clinical site personnel. These data can be used to scale this intervention, nationally.

## Organizations and study population

Beginning February 2014, *Expect With Me* was implemented in five clinical sites in Nashville TN, Detroit MI, and McAllen TX. Each site receives financial and advisory assistance to support the implementation and evaluation process. Yale and UnitedHealth Group recruited clinical sites. An important goal was to include sites located across different regions with different target populations. Interested sites completed a site eligibility form/site profile. If an organization met criteria to participate, a site visit was conducted by the research team to discuss the program procedures and requirements for participation in the study in more detail. The inclusion criteria for health care organizations was as follows: sufficient obstetric patient volume to meet study recruitment goals; support within the institution to integrate group prenatal care into practice; willingness to implement *Expect With Me* according to protocol during the study period; participation in the evaluation study data collection activities; and intention to continue *Expect With Me* after the project period.

To collect organizational data, all clinic staff involved in implementing *Expect With Me* were asked to participate in the process evaluation. To collect extensive data on individual-level outcomes, patients who participate in *Expect With Me* are required to enroll in the evaluation study. We aim to follow more than 1,000 pregnant women through one-year postpartum. Inclusion criteria for patients are as follows: less than 24 weeks pregnant; no severe medical problem requiring individual care only, as determined by the clinical practice; ability to speak English or Spanish; and willingness to participate in the study. Staff at each clinical site explain the study to eligible participants, answer questions, and obtain informed consent. A two-to-one matched cohort of women receiving standard individual care at each clinical site will provide the comparison for the outcome evaluation (two individual care patients for each group care patient). The matched cohort inclusion criteria mirror the study inclusion criteria (e.g., receiving prenatal care at the same clinical practice, entered prenatal care <24 weeks gestation, not transferred out to a high-risk clinic, fluent in English or Spanish, entered prenatal care during the study timeframe). All procedures have been approved by the Yale University Institutional Review Board and Institutional Review Boards at each study site.

## Training and technical assistance

Training for *Expect With Me* is a multi-phase process that targets health system staff at several levels. These phases include: (1) readiness planning and change management; (2) organizational training; (3) facilitator training; and (4) ongoing technical assistance. For several months prior to the start of patient enrollment in *Expect With Me*, health system leadership met regularly with the study team to prepare and build excitement for this care innovation. A series of webinars were conducted and emails sent to prenatal clinic staff providing general overview of *Expect With Me* and the benefits of group prenatal care for practices and patients. A one-day interactive organizational training was held to further familiarize prenatal clinic staff—from front desk staff to medical assistants to nurses, midwives, and obstetricians—with *Expect With Me* and each person’s role in making group prenatal care a thriving part of their practice. This training prepared prenatal clinics to begin *Expect With Me* care*,* including group scheduling, talking to new obstetric patients about group prenatal care, and using the IT platform. Clinical staff who would lead *Expect With Me* groups (e.g., obstetricians, midwives, nurses, medical assistants) participated in a one- to two-day training focused on the development of group facilitation skills. Study staff monitor implementation at each site in real-time through the IT platform and offer technical assistance through regularly scheduled calls to provide ongoing support, share learnings across sites, and troubleshoot issues, as needed. Site visits are conducted to ensure fidelity and support implementation. In-person meetings with all participating sites allow administrators and clinical staff to share knowledge and experience with *Expect With Me*. Group discussions during these meetings provide insights into the implementation process within different clinical settings, and determinants of successful implementation.

## Data collection and measurement

Both quantitative and qualitative data are being obtained at the organizational and patient levels. Table 2 shows how each of the data collection strategies will be used to assess key study outcomes.

**Table 2:** Assessment of key study outcomes

Outcome	Definition	Measure	Data Collection Strategy
Outcome evaluation			
Effectiveness	Impact of *Expect With Me* on patient outcomes	Patient-level - Comparison of birth outcomes between *Expect With Me* patients and the matched cohort who received individual care	- Health record reviews - Patient surveys
Process evaluation			
Uptake	Extent to which providers refer patients to *Expect With Me* and deliver group prenatal care sessions	Organization-level - Number and proportion of providers referring patients for *Expect With Me* during the enrollment period - Number and proportion of providers delivering *Expect With Me* group prenatal care sessions	- Health record reviews
	Willingness of eligible patients to participate in *Expect With Me* and extent to which they are representative of the target population	Patient-level - Number and proportion of eligible patients enrolled in *Expect With Me* - Characteristics of *Expect With Me* participants - Reasons patients participate/do not participate	- Health record reviews - Online tracking system - Patient surveys and focus groups
Fidelity	Extent to which *Expect With Me* is implemented as planned	Organization-level - Conformity to the implementation strategy - Conformity to the implementation content - Amount of sessions/groups completed - Amount of content delivered per session - Clinic staff perception about *Expect With Me’s* content and implementation strategy - Satisfaction about implementation within the clinic Patient-level - Amount of sessions attended - Amount of information technology platform use	- Online tracking system - Session evaluations - Provider interviews and focus groups - Patient surveys and focus groups - Site visits
	Amount of the program received by patients		
Sustainability	Impact of *Expect With Me* on healthcare costs	Organization-level - Comparison of financial impact of birth outcomes on healthcare costs - Comparison of utilization of healthcare, including visit attendance, emergency department and neonatal intensive care unit utilization, postpartum long acting reversible contraception uptake, and associated costs - Satisfaction with *Expect With Me* group care and provider training on group care	- Health record reviews - Billing records - Provider focus groups - Provider surveys
	Opinion about *Expect With Me* and the implementation strategy		

### Organizational-level: online tracking system

The *Expect With Me* IT platform was developed to collect real-time program implementation data, including the following information about every *Expect With Me* group: location, facilitators, session dates and times, group composition (e.g., number, gestational age range, and sociodemographic characteristics of participants), and session attendance rates.

### Organizational-level: provider surveys and session evaluations

Group facilitators at each site are asked to complete a questionnaire at baseline and one-year later. Items assessed include provider: demographics, clinical training, previous experience with group care models (baseline only); attitudes about group prenatal care; and perceptions of barriers and facilitators to implementing and sustaining *Expect With Me*. Group facilitators also complete a session evaluation following each prenatal care session scheduled. Data captured by each session evaluation includes: whether or not the session occurred; if a session did not occur, why was it cancelled and the follow-up plan to ensure patients receive their prenatal care; if the session did occur, the topics covered during the group discussion, time spent on physical assessments, and an assessment of the group dynamics and factors that may have influenced attendance or delivery of the session as planned.

### Organizational-level: focus groups and in-depth interviews

Many factors can potentially influence providers’ implementation behaviors including characteristics of the innovation (e.g., complexity, compatibility and alignment with clinic policy, culture, current practices), social setting (e.g., societal norms about prenatal care), organizational context (e.g., support, resources), innovation strategies (e.g., training, reimbursement), patient (e.g., attitudes, adherence to recommended care), and the individual provider (e.g., skills, attitudes) [54, 55]. Focus groups and in-depth qualitative interviews will be conducted with 30–50 key informants (i.e., health care providers, support staff, administrators), until saturation is reached, to identify key factors associated with implementation behavior that can inform strategies to promote adoption, effective delivery, and sustainability of *Expect With Me*. In-depth interviews will last approximately 45 min and focus groups will last approximately 60 min and be audiotaped and transcribed.

### Organizational-level: site visits

During site visits, study team members conduct selective observations of key interactions in prenatal care for patients (e.g., enrollment, group sessions) as well as relevant meetings and other onsite activities. Following each visit, site teams participate in debriefing sessions. Reflections and observations will be synthesized in written form and further examined as part of qualitative data analyses.

### Patient-level: surveys

*Expect With Me* participants complete five surveys: baseline (second trimester), third trimester, birth, 6-months postpartum, and 12-months postpartum. Online survey technology provides standardized measurement, computer-controlled branching through complex questionnaires, automated consistency and range checking, and multilingual administration of questions. Studies with high-risk populations have concluded that computer administered surveys provide valid and more accurate reporting of high-risk behaviors [56–59].

The baseline survey is the most comprehensive and includes patient sociodemographic characteristics and medical history. Follow-up interviews are shorter and assess changes in health-related attitudes and behaviors, as well as prenatal care satisfaction, infant nutrition, and maternal and child health care utilization. Measures include items related to: general health behaviors (e.g., smoking and drinking, diet, physical activity), pregnancy-related attitudes and health (e.g., readiness for labor and care, weight gain and retention), sexual health and risk (e.g., sexual activity, condom use and self-efficacy, intimate partner violence), depression, stress, discrimination, social support, resilience, internet access and use, infant feeding practices (e.g., breastfeeding intention and behavior, introduction of solid foods), and health care system utilization (e.g., emergency room visits, pediatric visits). All of the survey measures were selected from previous research; most have been validated with similar clinical populations [18, 21].

### Patient-level: medical record review

Primary outcomes and other maternal and infant health-related data will be gathered via prenatal, labor and delivery, and postpartum medical record extraction. Record extraction for each patient will be completed after 12-weeks post childbirth and one-year postpartum. Data collected will include medical and reproductive health history, prenatal visit attendance and vital signs (e.g., weight and blood pressure), pregnancy complications and outcomes, delivery information, birth outcomes (e.g., gestational age, birth weight), any neonatal intensive care admission/length of stay, and postpartum healthcare.

### Patient-level: focus groups

Focus groups will be conducted at each site with patients who have delivered their babies. Approximately 40 women will participate. The focus groups will be facilitated by two study team members, last 60–90 min, and be audiotaped and transcribed. Topics covered will include perceptions of prenatal visits, services and support received from the health system, curriculum, IT platform, and impact of the program on family health and well-being.

## Analyses

Descriptive analyses of quantitative data will be performed. Women who participated in group prenatal care and those who received individual care will be matched on the basis of propensity scores. Propensity score matching is a statistical technique to correct for observable difference between the treated and non-treated group, thereby reducing selection bias and strengthening causal inferences, in observational studies [60]. We will use multiple imputation and maximum likelihood techniques to handle missing data and sensitivity analyses to assess the robustness of inferences about treatment effects to various missing data assumptions, as needed.

Generalized linear and logistic regression models will be used to analyze continuous (e.g., gestational age at delivery and birth weight) and dichotomous outcomes (e.g., preterm birth, low birth weight), respectively. Negative binomial regression models will be used to analyze count outcomes (e.g., neonatal intensive care unit days). Each model will include a main effect for treatment group (*Expect With Me* vs. individual care). Because many women who attend group prenatal care sessions also supplement with individual visits [61], adjusted models will control for number of individual visits, as well as contain predictor variables that are clinically meaningful to a specific hypothesis of interest.

Trajectories of change for outcomes that are repeatedly measured over time (e.g., weight) will be examined using linear and non-linear mixed effects modeling, with a random effect for subject. An optimal covariance structure will be selected based on the likelihood ratio test for nested models and based on the Akaike Information Criterion for non-nested models [62]. The main independent effects of time, treatment, and second-degree interactions of these predictors will be considered in each model, adjusting for other covariates as described above.

Multilevel mixed models will be used to identify factors that influence primary outcomes among *Expect With Me* patients, with the effect of site modeled as a random effect. A scale variable that will reflect a woman’s attendance level at prenatal care group visits will be created to evaluate whether there is a “dose–response” intervention effect. Structural equation models will be used to examine factors that may mediate the relationship between participation in group care sessions and improved outcomes.

Focus group and in-depth interview data will be entered into NVivo 10 (QSR International Pty Ltd, Australia) and analyzed using the constant comparative method [63]. This type of thematic analysis is useful for identifying, examining, and reporting patterns within the data [64]. Coding of textual data will occur in iterative steps, in which codes are defined and then refined during review of transcripts from successive interviews and focus groups. Study team members will independently code transcripts. Intercoder reliability will be assessed, joint sessions held to discuss discrepancies, and final codes assigned to observations by a negotiated, group process. We will employ best practices for qualitative research including searching for disconfirming evidence, interviewing multiple respondents at each site for triangulation, and maintaining a detailed audit trail to document analytic decisions [63]. The qualitative data will also be used to interpret and extend the quantitative data findings.

## Cost analyses

The evaluation will include cost analyses [65, 66] comparing *Expect With Me* to individual care, based on patient outcomes (e.g., neonatal intensive care unit admissions and length of stay, emergency department visits). Data from each clinical site’s billing and accounting systems will be used determine patients’ health care charges and actual costs (i.e., amount paid for care collected by the health care facility) including minimum, maximum, median, and interquartile range values of costs for the entire pregnancy through three months postpartum, as well as for the prenatal, childbirth, and postpartum periods separately.

Ordinary least squares (OLS) regressions will be used to test the association between type of care received and maternal and newborn health care costs. Regressions will control for maternal age, race, and other risk factors for adverse birth outcomes. Generalized linear models (GLM) estimates with a gamma distribution and logarithmic link will be used to confirm OLS findings. Alternative approaches to OLS models often are used to account for the long right tail of health care cost distributions. However, OLS results are easier to interpret than many alternative model results. Previous studies of Medicare spending and other health care costs indicated that OLS models perform as well and sometimes better than alternative expenditure models [67, 68].

Costs associated with the provision of group prenatal care from a practice perspective, including start-up and maintenance costs, will also be assessed to inform scalability. Estimates for replication costs will take into account different clinical practice and implementation characteristics.

## Sample size

Results from previous randomized controlled trials suggest that group prenatal reduces likelihood of delivering a preterm or small for gestational age baby by 33–34% [18, 21]. Based on these findings and the national preterm birth rate of 9.6% [4], we need a minimum of 1,768 study participants with one-third (*n* = 884) receiving group prenatal care to have power of .80 to detect differences at alpha = 0.05 with a one-tailed test. With a potential 10% drop out rate due to miscarriage, pregnancy complications and changes in life circumstances (e.g., geographic move), we will recruit 1,000 women, expecting to follow more than 900 of them through one-year postpartum. We will compare primary maternal health and birth outcomes for these women with a 2–1 matched cohort of 2,000 patients receiving individual care. Therefore, the total study sample of women receiving both group and individual prenatal care will include a total of 3,000 women.

## Trial status

This study has been registered on ClinicalTrials.gov as NCT02169024. All data collection will be completed by December 2017.

## Discussion

Combined maternal and newborn care is the most common and expensive category of hospital care for all payers costing $52 billion for Medicaid and $40 billion for private insurers, annually [69]. High rates of preterm birth and low birth weight are important drivers of these costs. Moreover, gross disparities in these and other reproductive health outcomes are grounds for “evidence-based outrage” [70]. National initiatives to tackle disparities and rising rates of maternal morbidity and mortality [71–73] make a renewed focus on prenatal care access, structure, content and quality not only timely, but essential [74].

Group prenatal care has shown promise to reduce rates of adverse birth outcomes, particularly among African American women, but widespread dissemination of this important health care innovation has yet to realized. This paper describes the development and methods that are being used to comprehensively evaluate *Expect With Me*, an innovative model of group prenatal care with an integrated information technology platform designed to be scalable nationally.

Researchers, clinicians, health care administrators, insurers, and policy-makers will have significant interest in the results of this study if we can demonstrate that bundling prevention and care services into a *high-touch, high-tech* group prenatal care model (i.e., *Expect With Me*) meets the “triple aim” for maternity care: better healthcare quality, improved outcomes, and lower health care costs. Most importantly, families will avoid the emotional toll and lifetime health and education costs associated with adverse birth outcomes. Findings from this study will be used to formulate recommendations to optimize the dissemination and effectiveness of this model of group prenatal care.
